# Association of Microalbuminuria with Metabolic Syndrome among Aged Population

**DOI:** 10.1155/2016/9241278

**Published:** 2016-04-21

**Authors:** Xiao-Hong Li, Hai-Yan Lin, Shu-Hua Wang, Li-Ying Guan, Yi-Bing Wang

**Affiliations:** ^1^Health Management Center, Shandong Provincial Hospital Affiliated to Shandong University, Jinan, Shandong 250021, China; ^2^Department of Burn and Plastic Surgery, Shandong Provincial Hospital Affiliated to Shandong University, Jinan, Shandong 250021, China

## Abstract

*Background*. The impact of the various components of metabolic syndrome (MetS) on chronic kidney disease has been conflicting. We aim to investigate the association between MetS and microalbuminuria and identify the major contributing components of MetS that result in microalbuminuria in the Chinese aged population.* Methods*. A total of 674 adults aged 55–98 years (males: 266; mean age: 66.5 ± 7.5 years) were studied. MetS was defined by the 2004 Chinese Diabetes Society criteria and microalbuminuria by urine albumin-creatinine ratio (UACR) ≥3 mg/mmoL.* Results*. The prevalence of microalbuminuria was gradually increased with increasing number of MetS components (*P* < 0.05). In multivariate regression, after adjusting for age and sex, MetS was the strongest correlate of microalbuminuria (OR = 1.781, 95% CI = 1.226–2.587; *P* < 0.05) followed by the fasting plasma glucose (FPG) (OR = 1.217, 95% CI = 1.044–1.092; *P* < 0.05), systolic blood pressure (SBP) (OR = 1.011, 95% CI = 1.107–1.338; *P* < 0.05), and high-density lipoprotein cholesterol (HDL-C) (OR = 0.576, 95% CI = 0.348–0.953; *P* < 0.05).* Conclusions*. MetS is independently associated with microalbuminuria in the Chinese aged population. Elevated FPG is the most predominant component of metabolic syndrome associated with microalbuminuria followed by elevated SBP and reduced HDL-C.

## 1. Introduction

Microalbuminuria, defined as a urine albumin-creatinine ratio (UACR) of ≥2 g/mmoL, originally has been used as an early warning sign of chronic kidney disease and diabetic nephropathy [1]. Additionally, it has been known as a useful predictor of cardiovascular events in adults [2–4]. In light of these reports, it has been suggested that microalbuminuria screening should be added to the assessment of the cardiovascular disease risk profile, in addition to lipids and blood pressure [5, 6]. Recently, it has been declared that obesity was associated with increasing risk of renal injury [7].

Metabolic syndrome (MetS) is a clustering of metabolic abnormalities characterized by obesity, hypertension, dyslipidemia, and glucose intolerance that collectively increases the risk of diabetes mellitus, cardiovascular disease, stroke, and overall mortality [8, 9]. Epidemiologic studies suggested an independent association between MetS and microalbuminuria. However, studies on the association between various components of MetS and microalbuminuria, to some extent, were conflicting. Chen et al. [10], for example, reported a positive association between elevated BP, reduced HDL-C, elevated TG, and microalbuminuria in a cross-sectional study of US adults (aged ≥20 years). However, another national survey of 5659 adults aged 20–80 years in the US [11] revealed that among all MetS components BP and FPG demonstrated a strong association with microalbuminuria. Thus, further supporting evidence is needed.

In the present study, 674 Chinese people aged 55–98 years were observed to evaluate the association of MetS with microalbuminuria in a cross-sectional study. We aimed to investigate whether there is an independent association for MetS and microalbuminuria, as well as determine the critical elements of MetS that correlated with microalbuminuria in the Chinese aged population.

## 2. Methods 

### 2.1. Study Subjects

The data for this study was an electronic record of the enterprise population who underwent annual examinations between January and April of 2015 at the Health Management Center of Shandong Provincial Hospital affiliated to Shandong University, China. The electronic record form consisted of participants' general files (register code, name, age, sex, and register date), anthropometric files (body height, body weight, and blood pressure), laboratory files (blood and urine tests results), and other files (personal history of disease). With the exclusion of subjects of age below 55 years and subjects with missing data on UACR levels and body mass index (BMI), there were 674 subjects included in our analysis.

### 2.2. Study Measurement

The physical examination comprised blood pressure (BP) and anthropometric measurements, including height, weight, and BMI. BMI was calculated as weight (kg) divided by height (m)^2^. Well-trained nurses measured BP two times in the left arm of seated participants using an automated electronic device (OMRON model HEM-752 Fuzzy, Omron Company, Dalian, China) after 5-minute sitting. The average of the two readings was calculated to determine the reported BP. The venous blood samples were drawn after 12-hour overnight fasting for examining fasting plasma glucose (FPG), serum creatinine, triglyceride (TG), total cholesterol (TC), high-density lipoprotein cholesterol (HDL-C), and low-density lipoprotein cholesterol (LDL-C). Fasting 1st morning void urine specimens were obtained for determination of urinary albumin and creatinine. Urine albumin was measured using an immunoturbidimetric assay and urine creatinine using a kinetic assay using the Jaffe creatinine method. Elevated UACR in the current study was defined as UACR ≥3 mg/mmoL [12]. Glomerular filtration was calculated using the modified Schwartz equation: estimated glomerular filtration rate (eGFR) (mL/min/1.73 m^2^) = (0.413 × height in cm)/serum creatinine in mg/dL [13, 14].

### 2.3. Diagnosis of MetS

According to the 2004 Chinese Diabetes Society established criteria, the MetS was defined as having any three or more of the following factors: (a) overweight and/or obesity (BMI ≥ 25.0 kg/m^2^), (b) raised FPG (≥6.1 mmol/L) or previous diagnosis with type 2 diabetes and taking antiglycemic medication, (c) raised BP (≥140/90 mmHg) or previous diagnosis with hypertension and taking antihypertensive medication, (d) raised TG (≥1.7 mmol/L), and/or (e) reduced HDL-C (<0.9 mmol/L in men and <1.0 mmol/L in women) [15].

### 2.4. Statistical Analysis

Data analysis was performed by using SPSS 19.0 for Windows. The distribution of the different variables was examined and the appropriate parametric or nonparametric test was applied. Data are expressed as mean or percent and standard error. Variables with skewed distribution are expressed as geometric mean (95% confidence interval). Student's *t*-test was used to evaluate differences in mean and chi-square test to evaluate differences in proportions.

The study participants were divided into four groups according to the number of components of MetS. Anthropometric and laboratory features in each group were described, and *P*_trend_ across the groups was tested using linear regression analysis and Cochran-Mantel-Haenszel (CMH) for continuous and categorical variables, respectively.

A multivariate binary logistic regression model was used to find independent correlates of microalbuminuria. Adjusted odds ratios (OR) and 95% confidence intervals (95% CI) were calculated. Throughout analysis, *P* value <0.05 was considered statistically significant.

## 3. Results

### 3.1. Subjects' Anthropometric and Metabolic Characteristics by the Presence of MetS

The prevalence of MetS in the study was 31.2% for men and 28.4% for women. Anthropometric and metabolic characteristics of a total of 674 subjects (266 males and 408 females) are shown in Table 1. Mean age was 66.5 ± 7.5 years with 52.2% of 55–64 years and 47.8% of over 65 years. Compared to the subjects without MetS, participants with MetS had significant increases (except for total cholesterol) in age, BMI, systolic blood pressure (SBP), diastolic blood pressure (DBP), FPG, TG, LDL-C, and UACR levels (*P* < 0.05). There were also significant decreases in HDL-C and eGFR for those with MetS (*P* < 0.05).

### 3.2. Subjects' Anthropometric and Metabolic Characteristics by the Number of Components of MetS

**Figure 1:**
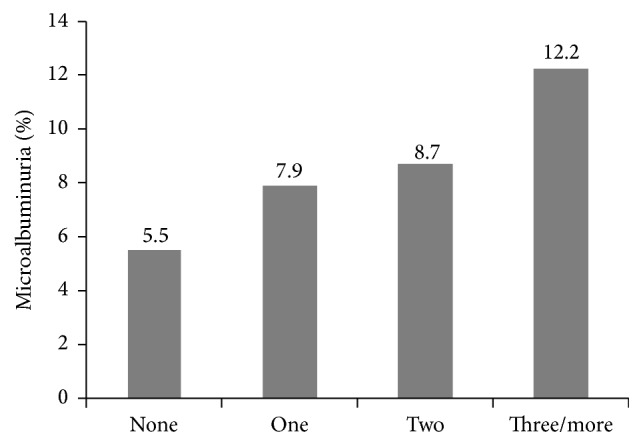
Prevalence of microalbuminuria according to the number of metabolic syndrome components.

The anthropometric and metabolic characteristics for participants with none (*n* = 134, NMS), one (*n* = 158, MS1), two (*n* = 183, MS2), and three or more (*n* = 199, MetS) components of MetS are shown in Table 2. With the number of MetS components, there were significant increases in age, BMI, SBP, DBP, FPG, TC, TG, LDL-C, and UACR levels (*P*_trend_ < 0.0001), whereas HDL-C and eGFR decreased (*P*_trend_ < 0.0001).  Figure 1 shows that the prevalence of microalbuminuria was significantly increased (5.5, 7.9, 8.7, and 12.2%, resp.) with the number of components of MetS (*P* values for linear trend <0.05).

### 3.3. Independent Correlates of Microalbuminuria

**Figure 2:**
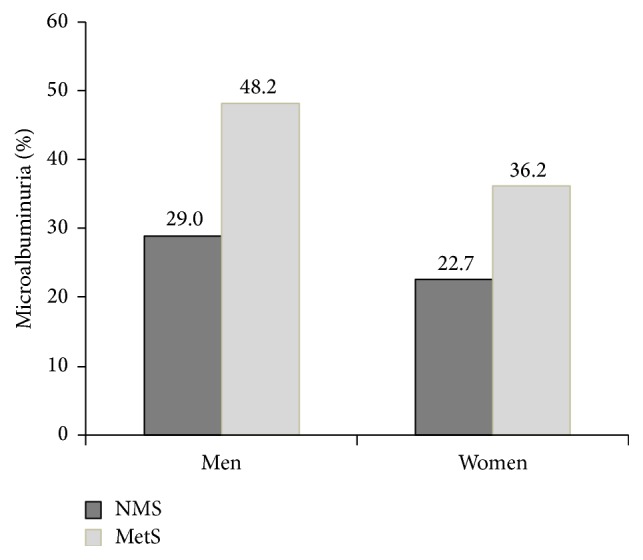
Prevalence of microalbuminuria according to metabolic syndrome.

The prevalence of microalbuminuria was 35.7% and 31.6% for men and women, respectively.  Figure 2 shows the prevalence of microalbuminuria in MetS group. In men, the prevalence of microalbuminuria was 48.2% in those with MetS and 29.0% in those without MetS (*P* < 0.05). In women, 36.2% of those with MetS had microalbuminuria, whereas 22.7% of those without MetS had microalbuminuria (*P* < 0.05).

Compared with those without microalbuminuria, the participants with microalbuminuria were older and had higher levels of SBP and FPG and lower levels of HDL-C and eGFR (*P* < 0.05) (Table 3). Based on these findings, SBP, FPG, HDL-C, eGFR, and MetS were used as potential correlates of microalbuminuria in a multivariate binary logistic regression model (Table 4). After adjusting for age and sex, MetS emerged as the strongest correlate of microalbuminuria (OR = 1.781, 95% CI = 1.226–2.587; *P* = 0.002), followed by FPG (OR = 1.217, 95% CI = 1.044–1.092; *P* < 0.0001), SBP (OR = 1.011, 95% CI = 1.107–1.338; *P* = 0.014), and HDL-C (OR = 0.576, 95% CI = 0.348–0.953; *P* = 0.032).

## 4. Discussion

It has been well documented that MetS is highly prevalent worldwide, especially in affluent countries [16–18]. Several previous studies have also reported the prevalence of MetS in the Chinese population. For example, the report in 2005 by Gu et al. based on a cross-sectional survey in a nationally representative sample of Chinese adults aged 35–74 years reported that the age-standardised prevalence of MetS was 9.8% in men and 17.8% in women [19]. In this survey, MetS was defined according to guidelines from the US National Cholesterol Education Program. Another report of 16,442 Chinese adults (aged ≥18 years) in 2010 indicated that the prevalence of MetS defined by the International Diabetes Federation (IDF) criteria was 23.2% (24.5% in men and 22.7% in women) [20]. In our study, the prevalence of MetS was 29.5% (31.2% in men and 28.4% in women). Although the reports differed in the study design and the targeted populations sampled, the higher prevalence of MetS in the current study, in conjunction with the higher prevalence of 34.1% (37.8% in men and 32.2% in women) reported in 2014 [21], as compared to the study in 2005 by Gu et al., may truly indicate a rapidly rising trend of MetS among the Chinese population over the 10-year period.

In the current study, the prevalence of microalbuminuria increased significantly with increasing numbers of MetS components after the participants were categorized according to the number of MetS components they had. The same result was obtained in a large study on 2,321 subjects in Japan [22]. The level of UACR was increased significantly even in the subjects with 1 or 2 components of metabolic syndrome. Therefore, it is suggested that earlier intervention for metabolic syndrome should be started to prevent the progression of chronic kidney disease.

The association between MetS and microalbuminuria has been shown in many studies [11, 23–27]. A study of Japanese people aged 40 to 87 years showed that, compared with those without MetS, those with MetS had a 1.99-fold (95% CI: 1.49 to 2.66) higher risk of microalbuminuria after adjusting for age and sex [22]. Another study of Korean adults aged 19 years or older reported that, compared with those without MetS, men and women with MetS had 3- and 2.79-fold higher risk of microalbuminuria, respectively, after adjusting for covariates [28]. However, the relationship between MetS and microalbuminuria has not been well elucidated in the Chinese aged population yet. In our study, we found that MetS was an independent and strong correlate of microalbuminuria in the aged population. Furthermore, MetS increased the risk of microalbuminuria 1.781-fold (95% CI: 1.226 to 2.587) after adjusting for age and gender.

Studies have yielded mixed results for the association between various fractions of MetS and microalbuminuria. For example, Hao et al. reported a positive association between high FPG, high BP, obesity, and microalbuminuria in a cross-sectional study of 2321 adults [22]. A study conducted on patients undergoing coronary angiography revealed among the components of MetS high FPG and lower HDL-C related to microalbuminuria [29]. Based on the impact of the various components of MetS on chronic kidney disease, the most recent study presented the concept of dividing all five components of MetS into a critical arm including elevated BP, reduced HDL-C, and elevated FPG and a noncritical arm including elevated TG and obesity [30]. Therefore, the impact of the components of MetS on microalbuminuria should be interpreted judiciously. Our results showed that elevated FPG was the most predominant component of metabolic syndrome associated with microalbuminuria followed by elevated SBP and reduced HDL-C. This result indicates that elevated FPB and SBP and lower HDL-C are likely to be critical components that lead a substantial number of subjects to the prestage of metabolic syndrome in the Chinese aged population.

Despite the previous reports, in our study, elevated SBP, not DBP, was associated with microalbuminuria. Indirect sphygmomanometric BP measurement is an established way of diagnosing and monitoring hypertension but overestimates the frequency of elevated DBP in the aged population [31]. Thus, the weaker association between the diastolic component of the arterial pressure and microalbuminuria in our study may be explained in part by the inclusion of subjects falsely labeled as hypertensive by the cuff sphygmomanometer in the aged population.

This study has some limitations. First, our population number was relatively small. Thus, prospective studies of a large sample should be conducted to verify the relationship between MetS and microalbuminuria. Second, the cross-sectional study design makes it hard to infer causality between MetS and microalbuminuria. Third, instead of waist circumference, BMI was used in the definition of MetS due to lack of corresponding data. This lack of data could have led to misestimation of the prevalence of MetS. Finally, ACR was determined on the basis of a single urine collection that participants were asked to collect in the morning of their visit. However, the high diagnostic accuracy of a single urine collection should minimise the uncertainty associated with day-to-day differences in the degree of albumin excretion [32].

In conclusion, the present study revealed a strong relationship between microalbuminuria and metabolic syndrome in the aged population in China. More comprehensive and intensive management of metabolic syndrome at its early stage should be started to prevent the progression of renal injury and cardiovascular complication.

## Figures and Tables

**Table 1:** Characteristics among participants with and without metabolic syndrome.

	No MetS (475)			MetS (199)			*P* value
	*n* (%)	Mean (SE)	95% CI	*n* (%)	Mean (SE)	95% CI	
Age		64.8 ± 5.4			67.9 ± 8.2		<0.0001
Male	183 (68.8)			83 (31.2)			0.035
BMI		23.9 ± 0.1			27.4 ± 0.2		<0.0001
SBP (mmHg)		130.8 ± 0.9			144.9 ± 1.3		<0.0001
DBP (mmHg)		70.4 ± 0.5			75.2 ± 0.9		<0.0001
FPG (mmol/L)		5.76	5.62, 5.90		7.21	6.86, 7.55	<0.0001
TC (mmol/L)		5.82 ± 0.05			5.98 ± 0.08		0.27
TG (mmol/L)		1.39 ± 0.04			1.62 ± 0.05		0.001
HDL-C (mmol/L)		1.43	1.39, 1.46		1.27	1.22, 1.31	0.001
LDL-C (mmol/L)		3.18 ± 0.04			3.48 ± 0.06		0.001
UACR (mg/mmol)		3.02	2.73, 3.31		5.36	4.36, 6.37	0.001
eGFR (mL/min/1.73 m^2^)		98.1 ± 0.9			92.0 ± 1.3		0.001

Data were means ± SD or medians (interquartile range) for skewed variables or proportions for categorical variables. Student's *t*-test was used to evaluate differences in mean and chi-square test to evaluate differences in proportions.

MetS: metabolic syndrome; SBP: systolic blood pressure; DBP: diastolic blood pressure; BMI: body mass index; FPG: fasting plasma glucose; TG: triglyceride; TC: total cholesterol; HDL-C: high-density lipoprotein cholesterol; LDL-C: low-density lipoprotein cholesterol; UACR: urinary albumin-creatinine ratio; eGFR: estimated glomerular filtration rate.

**Table 2:** Characteristics among participants according to the number of metabolic syndrome components.

	NMS (134)			MS1 (158)			MS2 (183)			MetS (199)			*P*_trend_
	*n* (%)	Mean (SE)	95% CI	*n* (%)	Mean (SE)	95% CI	*n* (%)	Mean (SE)	95% CI	*n* (%)	Mean (SE)	95% CI	
Age		63.2 ± 6.7			63.8 ± 5.6			66.2 ± 6.6			67.9 ± 8.2		<0.0001
BMI		22.2 ± 0.2			23.9 ± 0.2			25.1 ± 0.2			27.4 ± 0.2		<0.0001
SBP (mmHg)		121.8 ± 1.3			127.2 ± 1.3			140.7 ± 1.4			144.9 ± 1.3		<0.0001
DBP (mmHg)		67.0 ± 0.8			69.6 ± 0.9			73.7 ± 0.8			75.2 ± 0.9		<0.0001
FPG (mmol/L)		5.24	5.16, 5.31		5.58	5.40, 5.75		6.3	5.99, 6.61		7.21	6.86, 7.55	<0.0001
TC (mmol/L)		5.40 ± 0.06			6.00 ± 0.10			5.96 ± 0.09			5.98 ± 0.08		<0.0001
TG (mmol/L)		1.07	0.99, 1.14		1.43	1.30, 1.55		1.59	1.45, 1.72		1.62	1.53, 1.72	<0.0001
HDL-C (mmol/L)		1.52	1.45, 1.58		1.44	1.39, 1.50		1.35	1.30, 1.41		1.27	1.22, 1.31	<0.0001
LDL-C (mmol/L)		2.28 ± 0.04			3.31 ± 0.06			3.32 ± 0.06			3.48 ± 0.06		<0.0001
UACR (mg/mmoL)		2.89	2.37, 3.41		3.13	2.59, 3.67		3.02	2.55, 3.49		5.36	4.36, 6.37	<0.0001
eGFR (mL/min/1.73 m^2^)		100.0 ± 1.5			99.8 ± 2.0			95.6 ± 1.3			92.0 ± 1.3		<0.0001

Data were means ± SD or medians (interquartile range) for skewed variables or proportions for categorical variables. *P*_trend_ was calculated from CMH chi-square tests for categorical variables and linear regression analysis for continuous variables.

MetS: metabolic syndrome; SBP: systolic blood pressure; DBP: diastolic blood pressure; BMI: body mass index; FPG: fasting plasma glucose; TG: triglyceride; TC: total cholesterol; HDL-C: high-density lipoprotein cholesterol; LDL-C: low-density lipoprotein cholesterol; UACR: urinary albumin-creatinine ratio; eGFR: estimated glomerular filtration rate.

**Table 3:** Characteristics among participants with and without elevated UACR.

	UACR < 3 mg/mmol (450)			UACR ≥ 3 mg/mmol (224)			*P* value
	*n* (%)	Mean (SE)	95% CI	*n* (%)	Mean (SE)	95% CI	
Age		64.2 ± 6.3			68.4 ± 7.6		<0.0001
BMI		25.0 ± 0.2			25.1 ± 0.2		0.427
SBP (mmHg)		132.4 ± 0.8			140.7 ± 1.5		<0.0001
DBP (mmHg)		71.5 ± 0.5			72.7 ± 0.9		0.317
FPG (mmol/L)		5.8	5.69, 5.98		6.7	6.40, 7.07	<0.0001
TC (mmol/L)		5.9 ± 0.1			5.8 ± 0.1		0.279
TG (mmol/L)		1.43 ± 0.04			1.51 ± 0.05		0.087
HDL-C (mmol/L)		1.41	1.38, 1.45		1.35	1.30, 1.39	0.009
LDL-C (mmol/L)		3.28 ± 0.04			3.22 ± 0.05		0.633
eGFR (mL/min/1.73 m^2^)		97.8 ± 0.9			94.5 ± 1.3		0.005
MetS (%)	117 (26.0)			82 (36.6)			<0.0001

Elevated UACR was defined as a UACR ≥3 mg/mmol. Data were means ± SD or medians (interquartile range) for skewed variables or proportions for categorical variables. Student's *t*-test was used to evaluate differences in mean and chi-square test to evaluate differences in proportions.

MetS: metabolic syndrome; SBP: systolic blood pressure; DBP: diastolic blood pressure; BMI: body mass index; FPG: fasting plasma glucose; TG: triglyceride; TC: total cholesterol; HDL-C: high-density lipoprotein cholesterol; LDL-C: low-density lipoprotein cholesterol; UACR: urinary albumin-creatinine ratio; eGFR: estimated glomerular filtration rate.

**Table 4:** Binary logistic regression to find independent correlates of microalbuminuria.

	Unadjusted		Age- and gender-adjusted	
	OR (95% CI)	*P* value	OR (95% CI)	*P* value
SBP	1.019 (1.010, 1.027)	<0.0001	1.011 (1.107, 1.338)	0.014
FPG	1.257 (1.142, 1.383)	<0.0001	1.217 (1.044, 1.092)	<0.0001
HDL-C	0.556 (0.348, 0.886)	0.013	0.576 (0.348, 0.953)	0.032
eGFR	0.986 (0.976, 0.996)	0.005	0.993 (0.981, 1.005)	0.252
MetS	2.061 (1.437, 2.956)	<0.0001	1.781 (1.226, 2.587)	0.002

MetS: metabolic syndrome; SBP: systolic blood pressure; FPG: fasting plasma glucose; HDL-C: high-density lipoprotein cholesterol; eGFR: estimated glomerular filtration rate.
